# Our New York Letter

**Published:** 1890-07

**Authors:** Wm. L. Russell

**Affiliations:** New York


### Correspondence.

### OUR NEW YORK LETTER.

NEW YORK, June 12, 1890.

When the average minimum daily temperature reaches 60 degrees F. for several successive days, the "summer" diarrhœa of infants makes its appearance. It makes little difference whether the maximum temperature be excessively high or not. These views were advanced by Dr. A. Seibert, Professor of Diseases of Children, at the Polyclinic, in an article published in the *Medical Record* about two years ago, and have, I believe, been generally accepted here. The explanation of this relation between the degree of temperature referred to and the diarrhœa of infants is to be found, according to Dr. Seibert, in the fact that at this point milk readily begins to turn, and is fed to many children in that condition. The importance of this can hardly be overestimated, when it is added that considerably over eighty per cent. of the cases occur in infants under two years of age, whose diet consists almost entirely of milk.

The preservation of this food in hot weather has thus come to be regarded with special interest, and much labor has been spent in devising means to this end. The method of steaming is most popular here at present. It was discussed at the last meeting of the County Society, and a number of steamers or sterilizers were exhibited. Dr. A. Caillé, who opened the discussion, illustrated the practical efficiency of the method by exhibiting a bottle of milk closed with a plug of cotton, and which had been prepared in January. The milk was still perfectly sweet.

Dr. D. W. Chapin alluded to the apparatus known as the Arnold Automatic Steam Cooker, as the ideal steamer. The cost of this is about two dollars and a half.

Dr. H. Koplik showed a steamer devised by him for preparing milk, which he sold to his dispensary patients. It consisted of an ordinary tin wash boiler, around the sides and upon the cover of which was fastened a thick layer of felt to prevent radiation. About two inches of water were put into the boiler, and above the water was placed a perforated platform to hold the bottles. These bottles were first washed in hot water and soda, then in alcohol. They were now placed in an oven and heated in dry air, at a temperature of 130 to 180 degrees F., for fifteen minutes. Thus prepared, they were filled with milk, and without being corked, placed on the platform in the boiler. The cover was then put on, and heat applied by a gas or oil stove. In fifteen minutes each bottle was removed in a cloth, tightly corked with a rubber cork and returned. The cover was re-applied, and the steaming continued for forty minutes. Milk thus prepared, would keep perfectly for twenty-four hours at least, provided it were in good condition to begin with. Dr. Koplik added that absorbent cotton made the best cork. It should not be folded, but stuffed into the neck of the bottle without a fissure anywhere. It thus adapted itself to the irregularities of the glass.

Several other very simple varieties of apparatus were shown. Among them a simple perforated plate of tin, on which spools were fastened as legs, so that it could be placed in an ordinary covered pot. On this the milk could be placed in nursing bottles, closed with absorbent cotton stoppers.

By such simple means milk can be sterilized in any household, or by any dealer, and one great source of danger to hand-fed babies will be avoided.

Breast-fed babies are of course not exposed to the danger of spoiled milk, and diarrhœa is not so prevalent among them. In an analysis of three thousand cases made by Dr. F. M. Crandall, in a paper read before the American Pædiatric Society, which met here last week, the number of breast-fed babies was shown to have been only between seven and eight per cent. of the whole number, and in these the feeding was not regulated, and cleanliness of the nipple was not regarded. Dr. Crandall found that in regard to the comparative value of alkalies and acids, the former were of most use in the recent cases, and the latter in those of long standing.

In discussing this paper, Dr. J. H. Fruitnight, in referring to the use of antiseptics, said that all recommended had been thoroughly tried at the seaside hospital of St. John's Guild, to which large numbers of very bad cases from the tenement districts of the city were sent every summer, and they had not borne out the theoretical expectations. He added that at this hospital very little medicine was given, and that cases did wonderfully well under the combined influence of sea air, three or four sponge baths of alcohol and water every day, and regulation of the diet.

Dr. L. Emmett Holt, of the Polyclinic believed that the diarrhœa of children could be divided into two classes; one in which there were no essential pathological lesions, the other in which there were. The point of differentiation between these classes was the temperature; not the high temperature of the first days but the fever which continued for a considerable time. He thought that treatment by drugs was a failure. It was impossible to control intestinal fermentation in this way; the only drug which he believed to have any power being bismuth in large doses, two drachms to half an ounce daily. On the other hand drugs often did harm, especially the vegetable astringents, the tannin of which interferes with stomach digestion. He had found that milk peptonized by the Fairchild tubes,containing five grains of pancreatin and fifteen grains of bicarbonate of soda, was of great use, and that children did not object to the taste. Another useful measure was intestinal irrigation with a solution of common salt, a teaspoonful to a pint of warm water, which should be injected into the bowel by means of a long rectal tube attached to a fountain syringe.

At the April meeting of the County Society, Dr. Chapin stated that in some cases of summer diarrhœa milk was not tolerated in any form. In such cases it should be stopped entirely for twenty-four or forty-eight hours, and the child sustained on expressed beef-juice diluted with five times as much water, or with white of egg in water or barley-water. He also spoke favorably of stomach washing in some cases, a measure warmly advocated by Professor Seibert, and often successful in arresting vomiting when all other means had failed. The drugs used by him were small doses of calomel to begin with, bismuth, bicarbonate of soda, and sometimes dilute hydrochloric acid. Opium was occasionally necessary. The use of stimulants should be begun early.

At the same meeting Dr. J. E. Winters, Professor of Diseases of Children at the University, spoke of the importance of the management of the dietary. Most cases were preceded by indigestion, and some modification of the diet was necessary. Usually the quantity required reducing, the milk to be properly diluted and administrated sufficiently slowly and at proper intervals. Barley-water was a good diluent, and the addition of an alkali was desirable. When the diarrhœa was caused by fermented milk, it was ushered in by an attack of vomiting, purging, and some emaciation. In such cases the milk must be stopped, and some substitute given. His custom was to order equal parts of strong mutton broth and barley-water. This should only be continued for a short time, however, when an attempt should be made to resume the milk. It was sometimes best to give no food whatever for a short time until the stomach was emptied. Further treatment consisted in keeping the patient absolutely at rest and comfortably warm, in the administration of whisky, and if the intestinal irritation persisted, in giving opium. This drug must be used cautiously and never in repeated small doses. He also recommended intestinal irrigation. Professor J. Lewis Smith remarked that he directed his patients to use only milk which had been bottled in the country. This was steamed by placing the bottle on an inverted teacup resting in a kettle half full of water which was kept near the boiling point for two hours. He thought it best to discontinue milk entirely for a day at the beginning of the attack, beef-juice, barley-water, etc., being substituted. The medicinal treatment consisted of the use of bismuth and pepsin, with chalk mixture between the feedings. The best form in which to give opium was as paregoric which was comparatively safe, and could be given in doses of seven or eight drops every three hours, to a child of five months, or fifteen drops to one a year old.

The use of alcohol in the diseases of children was one of the subjects discussed by the Pædiatric Society. Dr. Seibert opened the discussion. He stated that as alcohol interfered with digestion, he never administered it by the mouth in disease of the stomach or in the acute diseases, while it was possible to nourish through the stomach. In diphtheria, for instance, he gave alcohol by rectum so long as food could be given by the stomach. The form of alcohol which he preferred was rye whisky. In infectious diseases he believes in beginning early and increasing the dose gradually and carefully, being guided more by the condition of the child than by its age or weight. A pint of rye whisky or Cognac in twenty-four hours for every hundred pounds of weight could be regarded as the maximum dose in acute diseases.

Dr. Winters said that no definite rules could be laid down regarding the time to use whisky; each case must be a rule unto itself. Alcohol was seldom necessary in labor pneumonia, except during convalescence. In broncho-pneumonia it should not be used until after the inflammatory stage had subsided. When, however, the superficial veins were full and the patient cyanotic it must be given early and freely. Also, in cases where the cough and other symptoms having subsided, it seemed as though the child was improving, but the physical signs persisted or extended; in such cases alcohol should be given in full doses. When there was great restlessness and sedatives failed, alcohol sometimes brought relief. If, however, local spasms or delirium, and injection of the conjunctival vessels were added, alcohol was contraindicated. He seldom employed it in infectious disease except diphtheria, in which disease he was guided by the condition of the circulation and the nervous system.

Professor Jacobi remarked that to use alcohol when there were symptoms of brain disease was simply shocking. It was also contraindicated in kidney diseases, whether acute or chronic. It was useful, however, in protracted convalescence, and in anæmia as a stimulant to digestion. In infectious disease too, large doses were often necessary to stimulate the heart. In diphtheria one could not give too much, and it should be begun before heart failure appeared, as afterwards it was too late. In scarlet fever with tendency to brain disorder it should not be used.

Dr. J. L. Smith said that he had lately used alcohol in a case of cerebro-spinal meningitis with great prostration, and asked Dr. Jacobi what he would have done under such circumstances. The latter replied that the case referred to was, he thought, exceptional, but he would prefer to try caffeine, camphor, digitalis, or sparteine instead of alcohol.

WM. L. RUSSELL.

